# Considerations on expanding criminal offender DNA databases with Y-STR profiles

**DOI:** 10.1093/jlb/lsae017

**Published:** 2024-09-04

**Authors:** Arwin Ralf, Martin Zieger, Manfred Kayser

**Affiliations:** Department of Genetic Identification, Erasmus MC University Medical Center Rotterdam, Rotterdam 3015 GD, the Netherlands; Institute of Forensic Medicine, University of Bern, Bern 3008, Switzerland; Department of Genetic Identification, Erasmus MC University Medical Center Rotterdam, Rotterdam 3015 GD, the Netherlands

**Keywords:** criminal offender DNA database, European law, familial search, human rights, sexual assault, Y-STR, RM Y-STR

## Abstract

Although national criminal offender DNA databases (NCODDs) including autosomal short tandem repeats (STRs) have been a successful tool to identify criminals for decades in many countries, yet there are many criminal cases they cannot solve. In cases with mixed male–female samples, particularly sexual assault, expanding NCODDs with Y-chromosomal STR (Y-STR) profiles allows database matching in the absence of autosomal STR profiles. Although Y-STR matches are not individual-specific, this can be largely overcome with rapidly mutating Y-STRs (RM Y-STR) allowing separation of paternally related men. Expanding NCODDs with Y-STR profiles is also beneficial for law enforcement in cases without known suspects via familial searching. Expanding NCODDs with Y-STR profiles may raise concerns about genetic privacy and fundamental human rights. A legal analysis of the European Convention on Human Rights revealed that when primarily for reidentifying convicted sex offenders, it would be in line with the case law of the European Court of Human Rights, while a generalized approach primarily for familial searching and involving all types of offenders may not. This paper aims to stimulate a debate among various stakeholders regarding the benefits and risks of expanding NCODDs with Y-STR profiles that in some countries has already been practically implemented.

## I. INTRODUCTION

For decades, short tandem repeats (STRs) located on the non-recombining portion of the Y chromosome (Y-STRs) are applied in forensic casework.^1^ Thus far, their main forensic application is when imbalanced male–female DNA mixtures with an excess of female DNA are encountered in crime scene material.^2^ Even though Y-STRs bear large forensic potential whenever a crime scene trace with an imbalanced mixture of male and female DNA is recovered, in practice Y-STR profiles are mostly generated in sexual assault cases. In such DNA mixtures, autosomal STR profiling of the male minor contributor often fails because of preferential amplification of the major female component and allele overlap with the female contributor. Even with knowledge about the autosomal STR profile of the female victim, as available from victim’s reference sample analysis, often there is insufficient evidential value to identify the male perpetrator from the mixed autosomal STR profile obtained from the trace.^3^ This can include cases where the same male perpetrator was previously convicted and therefore is included with his autosomal STR profile in the national criminal offender DNA database (NCODD), but cannot be re-identified because his autosomal STR profile could not be obtained from the mixed crime scene sample collected in the recurrent case.

In principle, this problem can be overcome by expanding NCODDs with Y-STR profiles. However, because in most countries, NCODDs solely consist of autosomal STR profiles, database matching based on Y-STR profiles currently is only rarely performed. Therefore, in most countries, the use of Y-STRs in forensic genetics is limited to cases with known suspects, who therefore are available for Y-STR reference sample testing for direct matching with the Y-STR profile from the crime scene. In many cases, however, case suspects are not yet known to the police at the start of the investigation, which is the prime reason why NCODDS were established in the first case in many countries already decades ago, albeit for autosomal STRs.

The second reason NCODD-based autosomal STR profile matching can fail is that the crime scene sample donor may not be included in the NCODD, having never been convicted of a criminal offense. In such cases, provided appropriate legislation, NCODDs can be used for familial searching, i.e., to identify a relative of the unknown perpetrator who, in contrast to the perpetrator, is included in the database. However, using NCODDs based on autosomal STRs for familial searching is restricted to very close relatives of unknown perpetrators, as consequence of DNA recombination. This results in a smaller pool of suitable relatives, thus a smaller chance that a relative practically is in the NCODD. Moreover, familial searching using autosomal STRs can lead to false positive results, haploid markers such as Y-STRs and mitochondrial DNA can be used to detect and exclude such false positive results.^4^ The lack of recombination of male-specific Y-STRs, as all forensically used Y-STRs are, leads to paternally related males often sharing the exact same Y-STR allele combinations (i.e., the same Y-STR haplotype), when using standard Y-STRs characterized by moderate mutation rates.^5^ Such haplotype sharing based on standard Y-STRs is typically observed among male relatives regardless of how close or distantly related they are.^6^ Sharing of Y-STR haplotypes between many male relatives largely improves the efficiency of familial searching compared with autosomal STRs. The more relatives can be highlighted, the larger the chance that one of those relatives is registered in the NCODD. However, because Y-STRs are not yet included in NCODDs in most countries, their use for familial search is restricted to the types of forensic cases without known suspects that do not rely on NCODDs, such as disaster victim identification, missing persons cases, and dragnets or mass screenings such as in cold case investigations.^7^ Moreover, Y-STR-based familial searching is only effective if a male is involved and another patrilineally related male is present in the database. Hence, familial searching using autosomal STRs would continue to be relevant and both approaches should be regarded as complementary.

Despite the forensic suitability and relevance of Y-STRs, in the bulk of forensic cases, only autosomal STR profiling is done followed by NCODD matching to determine if the STR profile from the crime scene sample is completely identical to the STR profile of a known offender in the NCODD. The main purpose of NCODDs, and the reason why they were established in the first case, is to efficiently re-identify previously convicted criminal offenders in cases where they commit subsequent crimes.^8^ Moreover, NCODDs also serve the purpose of linking different crime cases involving the same perpetrator. Recovering the same autosomal STR profile from crime scene samples of different criminal cases implies that the same perpetrator was involved in these different cases. This broadens the search to find the perpetrator via NCODD matching and allows solving several cases at once as soon as a database match is achieved. Notably, just as important as the identification of crime scene sample donors is the exclusion of innocent individuals who are wrongly suspected in the course of an investigation by demonstrating that their reference STR-profile does not match that of the crime scene sample. The fact that male relatives often share the same Y-STR profile lowers the evidential value of a Y-STR match for identification. Nevertheless, a non-matching Y-STR profile in a crime scene trace holds similar evidential value for exclusion as a non-matching autosomal profile.

With the help of NCODDs based on autosomal STRs, thousands of criminal cases are solved globally each year. Moreover, NCODDs and DNA evidence in general have also led to many wrongfully convicted individuals being exonerated.^9^ NCODD-based identifications not only occur within a country by using the local NCODD, but also across countries, e.g., those EU Member States that are part of the treaty of Prüm, based on which forensic STR profiles are cross-searched between NCODDs of member countries to fight cross-border crimes.^10^ There are two key factors that generally make or limit the success of using NCODDs for identifying perpetrators of crime. First, the DNA profile needs to be of sufficient quality to allow NCODD searching and eventual matching. Second, to allow the match with the DNA-profile from the crime scene sample, the NCODD must contain the DNA profile of the searched crime scene sample donor. Notably, not all criminal cases where potentially informative DNA traces are collected can be solved with autosomal STRs as pointed out above, which can partially be overcome by expanding NCODDs with Y-STR profiles.

From a law enforcement perspective, complementing NCODDs with Y-STR profiles offers several advantages for cases involving known male suspects as well as for those with unknown male perpetrators.^11^ For cases with known male suspects included in the NCODDs, which cannot be identified in other cases because no autosomal STR profile could be obtained because of male–female DNA mixtures, a NCODD match based on standard Y-STRs would highlight the individual and any of his (close or distant) male relatives if such relatives are included in the NCODD. Subsequently, the use of non-standard Y-STRs with elevated mutation rates, such as rapidly mutating Y-STRs (RM Y-STRs),^12^ can exclude relatives of the true perpetrator provided that a mutation has occurred in at least one of the familial lines for at least one Y-STR, which is likely for most types of relatives.^13^ Conversely, RM Y-STRs can help establish that the suspect indeed is the sample donor in cases when the person in the NCODD has the same RM Y-STR haplotype that is found in the crime scene DNA. For cases with unknown male perpetrators not included in the NCODD, any close or distant patrilineal male relative included in the NCODD will likely deliver a match based on standard Y-STRs. RM Y-STRs can then help providing investigative leads to find the branch of the lineage to which the unknown donor of the crime scene sample belongs, with less allelic differences with closely related males and more with distantly related ones. Typically, from a policing perspective, close relatives provide stronger investigative leads than distant ones. Thus, when it comes to Y-STRs in the NCODD, the same concept based on a sequential procedure of applying standard Y-STRs and RM Y-STRs applies to both type of cases, with and without known suspects.

Over the years, the number of Y-STRs included in commercially available Y-STR kits has constantly risen with only six markers in the first commercial Y-STR kit 20 years ago to 22 to 25 Y-STR markers in the latest generation of commercial Y-STR kits (because such kits include multi-copy Y-STRs, where the different copies cannot be separated with the PCR primers used, the number of Y-STR loci they target is higher than the number of Y-STR markers they include).^14^ This expansion of the number of targeted Y-STR markers and loci has enhanced the kits’ discriminating capacity, allowing for improved differentiation of unrelated men from different male lineages in many populations.^15^ The necessity for expanding commercial Y-STR kits with more markers was mainly motivated by population studies demonstrating that with previous generation Y-STR kits harboring less markers, unrelated males shared the same Y-STR haplotype by chance (identity-by-state).^16^ Moreover, the proportion of haplotype sharing differed between populations from different parts of the world because of differences in male population history.^17^ While commercial Y-STR kits contain standard Y-STRs with moderate mutation rates (in the order of one or a few mutations every 1000 generations per each marker), and were mostly expanded with standard Y-STRs, some later generation kits additionally included RM Y-STRs, albeit in much smaller numbers than are available outside these commercial kits. RM Y-STRs with their increased mutation rate (one or a few mutations every 100 generations per each marker) increase the differentiation of unrelated men and at the same time allow differentiating related males with increased chance the more distantly related the male relatives are.^18^ Moreover, by using more RM Y-STRs than the few currently included in the latest generation commercial Y-STR kits, the ability to separate closely related males strongly increases.^19^ The recent non-commercial RMplex kit,^20^ which includes 30 Y-STRs with elevated mutation rates including all 26 currently known RM Y-STRs, provides differentiation rates of 43% for father-sons, 66% for brothers, 83.5% for first cousins, and even higher rates up to 100% for more distantly related men. Notably, these male relative differentiation rates established with RMplex were significantly higher compared to the ones obtained with one of the latest generation commercial Y-STR assay, the Yfiler™ Plus PCR Amplification Kit, which differentiated 10%, 22%, and 27% in the same familial relationships using the same samples, respectively.^21^

As mentioned above, to maximize the forensic value of Y-STRs, a combined approach utilizing both Y-STRs and RM Y-STRs is necessary. The process should begin with the analysis of standard Y-STRs, at best via latest generation commercial Y-STR kits, and inclusion of the resulting profiles in the NCODD. If a match is found, the next step should be typing with RM Y-STRs, at best with all available RM Y-STRs via commercial kits that currently are not yet available (while the non-commercial RMplex kit is available). The number of RM Y-STR differences, if any, will indicate the degree of relationship between the sample donor and his male relative in the NOCDD, with fewer differences for close and more differences for distant relatives. This information will provide investigative leads to find the unknown perpetrator via investigating his male lineage that was highlighted through the standard Y-STR haplotype match in the NCODD. The analysis of RM Y-STRs allows finding the branch of the family that is most closely related with the unknown sample donor, providing most useful investigative leads for identifying him.

## II. IDENTIFYING KNOWN OFFENDERS

The initial reasoning behind establishing NCODDs based on autosomal STRs was to allow re-identification of criminal offenders, who previously committed a crime for which they were previously convicted,^22^ as soon as they commit the next criminal offense. This represents a shortcut in identifying perpetrators with the help of the NCODD in cases of a subsequent crime. This shortcut is based on the well-established evidence that criminal behavior often is repetitive.^23^ Unfortunately, in many sexual assault cases involving male perpetrators who already are in the NCODD because of previous conviction, no autosomal STR profile of the perpetrator can be generated in the new case because of male–female DNA mixtures as outlined above. Given the seriousness of the crimes of rape and other sexual assault,^24^ and because sexual offenders also are repetitive – albeit apparently less often than other types of offenders,^25^ a valid line of reasoning is to complement NCODDs with Y-STR profiles, particularly for the reason of reidentifying sexual offenders.

### II.A. Sexual Assaults and NCODD Inclusion Criteria

In forensic practice, a focus on reidentifying sexual offenders via Y-STRs in the NCODD would be achieved by only generating Y-STR profiles from crime scene traces of sexual assault cases and only from references samples of convicted sexual offenders for inclusion in the NCODD. Under such selective scenario, when a crime scene trace obtained from a sexual assault case would not lead to a usable autosomal STR profile, a Y-STR profile is obtained from the evidence DNA sample, uploaded to the NCODD, and compared with all Y-STR profiles from all sexual offenders included in the NCODD to search for a Y-STR match. The disadvantage of such selective approach is that sexual offenders who are already included in the NCODD with their autosomal STR profiles because of a previous *non-sexual* offense, cannot be identified with Y-STRs, because their Y-STR profiles will not be included in the NCODD with such approach. Given the evidence that many sexual offenders have prior convictions, and a reasonably large number (e.g., 24% in a study from the US^26^) were convicted for non-violent crimes, this selective approach would fail at identifying many sexual offenders of which their autosomal STR profiles are included in the database, but their Y-STR profiles are not due to the use of such selective approach. The same applies to previously unidentified sexual offenders that are later convicted for a non-sexual offence. When Y-STR profiles of non-sexual offenders are not included in the NCODD, the link of such recidivists to the prior sexual offence cannot be detected. The notion that sexual offenders tend to commit non-sexual offenses is supported for instance by a study that investigated the records of 3070 men that were convicted for sexual offences in the year 1973 in England and Whales. In the period from 1963 to 1994: 45% of those males were also convicted for theft and handling in stolen goods, 24% for burglary and 24% for violence against a person, over 60% were convicted for a non-violent offence in that time period.^27^ These data suggest that a significant portion of men that commit sexual offences are also involved in other types of crimes. Given the large impact sexual crime has, for victims of sexual offences and their families and for society, it is of great importance that the perpetrators of sexual crime are held responsible for their actions and to discourage them from continuing.^28^ Limiting the inclusion of Y-STRs in the NCODD to convicted sexual offenders will result in some sexual offenders escaping the consequences of their sexual offence and can lead to their continuation of committing sexual offenses.

To overcome this limitation and increase the effectiveness of Y-STRs in a NCODD to reidentify sexual offenders, Y-STR profiles would need to be generated from reference samples of *all* criminal offenders of *all* types of crimes that qualify for entry in the NCODD, sexual and non-sexual offenses, and registered in the NCODD. In practice, the focus on sexual assault cases can still be maintained by only generating Y-STR profiles from crime scene samples in cases of sexual assault where no usable autosomal STR profile is obtained, and thereby restricting the NCODD search to Y-STR profiles in cases of sexual assault. The restriction of using the Y-STR-based NCODD on cases of sexual assault would be assured also under this scenario, because in cases of non-sexual assault, Y-STR profiles would not be generated from the crime scene sample and Y-STR searches in the NCODD will therefore not be carried out. The latter would not be necessary anyway, as in non-sexual assault cases, an autosomal STR profile of the sample donor would likely be available in case of single source (non-mixed) crime scene material, which allows NCODD matching based on autosomal STR profiles. However, imbalanced male–female mixtures can also be observed in non-sexual types of violent crimes. For example, under an activity scenario where a female victim scratched her male attacker aiming to defend herself and consequently, low-levels of his DNA may be present under her fingernails together her own DNA.^29^

### II.B. The Evidential Value of a Y-STR Match

Regardless of the chosen NCODD inclusion criteria applied for Y-STR profiles, using Y-STRs for the purpose of individual identification is challenging as typically a matching haplotype based on standard Y-STRs would point to a male lineage rather than a single male individual. However, this can be overcome to a large degree by use of RM Y-STRs, as outlined above. Ongoing activities to discover more RM Y-STRs and demonstrate their value to further improve male relative differentiation, or the use of Y-chromosome markers other than RM Y-STRs, are expected to furher improve the ability to difference between male relatives based on Y-chromosome DNA analysis in the near future. At the very least, using highly discriminatory Y-chromosome markers such as RM Y-STRs and others can substantially narrow down the number of individuals within a male lineage that would match to the crime scene stain. In the ideal scenario, RM Y-STRs alone or together with other Y markers, exclude all other known relatives in a male lineage by showing allelic differences compared with the Y-chromosome profile from the suspect and the matching crime scene trace. Such Y-chromosome differences are then explained by mutations between the related men, except for one single matching male, who is the crime scene sample donor. An example can be found in the Norwegian case of the rape and murder of Birgitte Tengs. In this case, it was shown that the Y-STR profile of the male suspect matched with the Y-STR profile from a crime scene sample, while the Y-STR profiles of his two brothers did not, due to RM Y-STR mutations. This excluded the two brothers from having donated the crime scene sample.^30^ In this case, all three brothers could be distinguished from one another based on RM Y-STRs. However, it is important to note that Y-STR mutations occur under are a largely stochastic process and that in other cases brothers may not be distinguishable with RM Y-STRs (see above for empirical male differentiation rates).

The possibility that a suspect’s male relative not being known to the police as result of the case investigation and therefore not involved in the RM Y-STR testing, could share the same RM Y-STR haplotype as the suspect, and therefore may be the true perpetrator, requires a careful characterization of the male lineage. This puts emphasis on police investigation to highlight all male relatives of the male suspect and request from them to participate in RM Y-STR testing for the purpose of exclusion. However, with the use of RM Y-STRs, the focus in the police investigation to find male relatives of the suspect has to be more on finding and testing *closely* related male relatives, than to find all relatives including distantly related ones. This is because the more distantly related a relative is, the more likely he will show RM Y-STR differences, which excludes such relative from being the potential sample donor. Typically, it is easier via police investigation to locate closely related men than distantly related ones, which provides an advantage when using RM Y-STRs. Moreover, many close relatives can likely already be excluded as potential perpetrators based on non-genetic information, hence mitigating the need for genetic methods of exclusion.

## III. IDENTIFYING UNKNOWN OFFENDERS

Another obvious limitation of NCODDs solely based on autosomal STRs is that perpetrators that have not been previously convicted, and are therefore not included in the NCODD, cannot be immediately identified, because their reference autosomal STR profile is missing from the NCODD. This has negative consequences for individuals becoming victims subsequently, families of victims and for society, for as long as the perpetrator does not get identified via other means and he can continue to commit more crimes. One way to overcome this is to perform familial searching in the NCODD, i.e., searching for individuals in the NCODD with similar autosomal STR profiles, allowing highlighting relatives of the unknown sample donor included in the database, while the sample donor is not in the NCODD. Familial searching in NCODDs using autosomal STRs is legally possible in several countries where it is allowed by law or at least not forbidden by law, depending on the law system in place (see chapter below). For instance, it is allowed and has been used successfully in the Netherlands in several cases, for example in one case to identify a man that had physically assaulted and murdered several homosexual men in Rotterdam.^31^ However, the reach of such an approach based on autosomal STRs is typically restricted to first- and second-degree relatives of the unknown perpetrator, which reduces the chance that one of such a low number of relatives is included in the NCODD. This is because of the relatively small number of forensically-used autosomal STRs and the process of DNA recombination, which reshuffles DNA, including the autosomal STR alleles in the offspring at every next generation. If Y-STRs would complement autosomal STR profiles in the NCODD, however, this would open-up the possibility of an extended familial searching approach within the NCODD, identifying close as well as distant male relatives of the unknown male sample donor, who are included in the NCODD. There are relatives that are identified when familial searching is carried out based on autosomal STRs in NCODDs, with increased success the larger the size of an NCODD is. There seems to be at least some evidence for an increased chance that sons of criminal offenders will be convicted compared with those whose fathers were never convicted.^32^ Such a potential link between criminal careers of fathers and sons would also lead to an increased chance to identify the criminal son when the convicted father is included in the NCODD, or the other way round. However, because in general more namely close and distant, relatives can be identified with Y-STRs, compared to less, namely only close, relatives with autosomal STRs used in forensics, the use of Y-STRs in the NCODD will increase the chance of success in familial searching in cases with unknown male sample donors.

In such a scenario, any type of crime scene material that did not result in an autosomal STR match would be used to generate a Y-STR profile. The obtained crime scene Y-STR profile would be compared to the reference sample Y-STR profiles in the NCODDs. A (near-) match with standard Y-STRs would point to a paternal relative of the sample donor, which can be further refined with RM Y-STRs. The number of allelic differences of RM Y-STRs between the crime scene profile and the individual included in the database is indicative for the degree of relationship between both individuals.^33^ Smaller number of RM Y-STR allelic differences indicates close relatives while larger numbers demonstrate distant relationships. From a practical perspective, cases where only a distant relative can be identified may not be further pursued as such an investigation could entail dozens or even hundreds of males. However, when a reasonably close relative (e.g., likely separated by no more than 10 generations) is found, this genetic knowledge together with genealogy information on the pedigree structure and tactical police investigations can then result in investigative leads that can ultimately uncover the identity of the sample donor. Here, Y-STRs would be used as an investigative tool and autosomal STRs will eventually be used to confirm if the person traced via this investigative Y-chromosome genetic approach indeed is the donor of the crime scene trace.

This approach could help solving cold cases, it could prevent future crime from occurring, and it may help to identify young offenders at an early stage, thereby potentially preventing them from committing more serious crimes at later age. However, proportionality should be considered here, as such an investigation would also include several innocent family members, it should only be considered for serious crimes and not be applied for minor offences. Notably, this approach is already applied in forensic practice, but mostly outside NCODDs and thus only in specific cases via case-specific voluntary mass screenings with enormous efforts, such as the rape and murder of Marianne Vaatstra in the Netherlands.^34^ If Y-STRs would be included in NCODDs, it can be expected that many cases will ultimately be solved via Y-STR database searches, and therefore mass screenings can be avoided in many cases. Only for severe cases that still cannot be solved via the Y-STR-based NCODD, because the sample donor himself and none of the unknown sample donor’s paternal male relatives are included in the NCODD, a dragnet approach, or other expensive and labor-intensive approaches such as investigative genetic genealogy via SNP microarrays or whole genome sequencing could be considered as a last resort.^35^

## IV. PRACTICAL CONSIDERATIONS

With more and more Y-STR markers included in commercial forensic Y-STR kits, and with the identification and forensic application of more RM Y-STRs and other Y-chromosome markers that can differentiate between male relatives, there is a need for better means to evaluate the evidential value of a Y-chromosome, such as Y-STR match. Currently, the use of population frequencies derived from unrelated men is recommended by the DNA Commission of the *International Society of Forensic Genetics* (ISFG) also for Y-STR haplotypes.^36^ However, given the very high discrimination capacity achievable with the last generation Y-STR kits, which would increase further with more RM Y-STRs being used, this approach may no longer be appropriate.^37^ The more diverse Y-STR haplotypes become, as they do by increasing the number of Y-STR markers and especially so when using RM Y-STR markers, the larger the population database needs to be to yield a somewhat reliable Y-STR haplotype frequency estimate. In reality, however, existing population databases contain more data for older generation Y-STR kits and smaller number for the newer generation kits.^38^ Because new generation commercial Y-STR kits typically contain the Y-STRs from the older generation kits, the data added to the population database for the new kits will increase the database content for the Y-STR haplotypes defined by the older kits, while for the expanded Y-STR haplotypes from the new kits, the database content starts at zero when a new kit is introduced. However, the haplotype frequency estimates achieved with the old kits still define a minimum frequency as the extended haplotype from the new kit will get rarer with additional Y-STRs, not more common, in unrelated men. Moreover, currently existing population frequency databases for Y-STR haplotypes consist of unrelated men only, while populations include unrelated and related men. Therefore, Y-STR haplotype frequency estimates from current population databases reflect underestimates, while the true frequency of a Y-STR haplotype in a population will be higher depending on the number of male relatives in a population.^39^

Arguably, a more suitable approach than using population frequency databases would be to focus on the specific male pedigree to which the suspect belongs, rather than the population as a whole. For example, the evidential value of a (RM) Y-STR match will be higher for a suspect who is a single son and whose father and paternal grandfather were single sons too, compared to a suspect who has many brothers and whose father and paternal grandfather came from large families with many sons too. Ideally, multiple non-suspected males from different branches of the pedigree would be characterized using highly discriminatory Y-STRs such as RM Y-STRs to exclude individuals and their branches as potential contributors and to infer the ancestral haplotypes that are found in the pedigree.

Whether Y-STR typing of these relatives should be on a voluntary or on a mandatory basis must be considered carefully. Non-participation in a voluntary approach may lead to suspicion and subsequently to the individual or his relatives being investigated. This leads to some pressure to participate voluntarily, what runs against the legal principle *nemo tenetur se ipsum accusare.* Men should not be asked to voluntary contribute to the collection of evidence that can be held against themselves, or their male relatives. Therefore, a mandatory participation in the context of specific case circumstances and with strict legal safeguards may be preferable.^40^

Characterizing multiple male relatives within a family is vital to make an accurate assessment of the evidential value of a Y-STR match. For example, if a suspect were to show a deep ancestral haplotype, this would lower the evidential value due to the increased chance that this haplotype will be shared by more relatives. If the suspect shows deviations from the ancestral haplotype and from his direct ancestors (e.g., from his father), the number of relatives that may share the same haplotype as the suspect will be strongly reduced. Future technological developments may enhance the ability to differentiate between close relatives even further. Notably, as of yet, no such method for pedigree-based establishment of the evidential value of a Y-STR match is available, but development is currently underway by some of the authors.

To minimize the burden of a familial search on the innocent male family members, it is of importance that the results of the investigation prior to genetic identification of a suspect are not made public. The authorities should therefore make an effort to perform the investigations in a discrete manner. Overall, while the inclusion of Y-STRs in NCODDs may lead to increased privacy concerns relative to autosomal STR (see below), it is important to balance these concerns against the potential benefits of solving crimes and preventing future crimes from occurring. The extra consideration when it comes to the use of Y-STRs in NCODDs is their increased familial component towards more distant relatives, which, however gets less and less the more RM Y-STRs are analyzed to differentiate these relatives. Perhaps, in the more distant future, Y-chromosome evidence can be established that allows identifying a single man solely based on Y-chromosome evidence. Moreover, other investigative genetic genealogy approaches using commercial databases rather than NCODDs are rapidly gaining popularity and have their own ethical, technical and practical dilemmas. Partly because these databases were not established for law enforcement purposes, even though participants nowadays can give informed consent for the use of their DNA data for criminal investigations, these systems are not flawless.^41^

Regarding advances in forensic DNA technology, over the recent years, targeted massively parallel sequencing (MPS) has been introduced in specialized forensic laboratories for various purposes including autosomal STR analysis.^42^ Targeted MPS has advantages also for autosomal STR profiling of DNA mixtures, including imbalanced male:female DNA mixtures. Iso-alleles that harbor sequence variations undetectable with capillary electrophoresis (CE) can be found with MPS leading to increased discriminating capacity.^43^ Most NCODDs, however, are not yet equipped to store the additional sequence information that can be obtained via MPS. While targeted MPS can also be used for Y-STR analysis, the most common mutations in Y-STRs cause changes in the length of the allele. Therefore, the added value of MPS in differentiation close male relatives compared to CE is limited. Moreover, several RM Y-STRs are longer than the maximum fragment length that can be sequenced with the ‘short read’ MPS technologies currently applied in forensic DNA analysis. Until long-read sequencing technologies are more established in forensic DNA analysis, PCR-based fragment length analysis with CE remains the DNA technology of choice for RM Y-STR analysis.

## V. COMPATIBILITY WITH FUNDAMENTAL RIGHTS

Expanding NCODDs with Y-STR profiles undoubtedly entails ethical implications. However, unlike research databases in genetics, NCODDs are less direct subject to international ethics guidelines. Rather, their admissibility must be assessed based on the prevailing fundamental rights framework.^44^ Such a framework, binding for its 46 contracting states, is established by the Council of Europe (CoE) through the European Convention on Human Rights (ECHR). In the following, we will explore the jurisdiction of its court, the European Court of Human Rights (ECtHR), to assess whether NCODDs based on Y-STR profiles could be considered in line with the ECHR.

### V.A. Scope of Article 8 ECHR

In its rulings concerning entries from individuals in databases serving the criminal justice system, the court repeatedly assessed the admissibility of the measure in question under the provisions of the ECHR in the light of its article 8. Article 8 of the Convention states:

1. *Everyone has the right to respect for his private and family life, his home and his correspondence.*
2. *There shall be no interference by a public authority with the exercise of this right except such as is in accordance with the law and is necessary in a democratic society in the interests of national security, public safety or the economic well-being of the country, for the prevention of disorder or crime, for the protection of health or morals, or for the protection of the rights and freedoms of others.*

The ECtHR repeatedly stated that the generation and storing of DNA profiles amounts to an interference with Article 8 of the Convention.^45^ In the case of a domestic law that is sufficiently specific and accessible, the Court will assess the admissibility of an interference with Article 8 for the legitimate aim of the detection and prevention of crime, according to the question whether such a measure is necessary in a democratic society. If we assume the principal suitability of the measure for achieving the intended goal, the Court will therefore assess the actual proportionality of it. To do this balancing, the Court will have to take into account the reasons for the storing of genetic data in the NCODD. Based on what we outlined in the previous chapters, here, we have to differentiate between (i) the purpose to detect and prevent recidivism of convicted sex offenders in case a potential suspect is registered on the NCODD and (ii) the purpose to facilitate familial searching in case a potential suspect is not registered on the NCODD.

### V.B. Registering Y-STR Profiles in NCODDs for the Purpose of Detecting and Preventing Recidivism

When it comes to the decision who ought to be included for the long term in a NCODD, the ECtHR seems to follow what has been called the ‘recidivism theory’.^46^ The prior conviction serves as justification for the data storage in the NCODD.^47^ The Court repeatedly differentiated between convicted offenders and mere suspects in this regard.^48^ It differentiated as well according to the seriousness of the offense, recalling the state’s duty to protect its citizens from grave inferences, namely such as sexual assault.^49^ This duty entails also the prevention of repeated offence by individuals who have already demonstrated of being capable to commit such violent crimes.^50^

Following the Courts ‘recidivism theory’ and taking into account the acknowledged state’s duty to protect, we can expect for good reasons that the ECtHR would consider a NCODD including Y-STR profiles with the purpose to detect and prevent recidivism as admissible before the law. The fact that registered Y-STR profiles can be used for familial searching does not prevent their registrations *per se*, as familial searching is as well possible, even though less efficient, with currently existing NCODDs based on autosomal STR profiles, as has already been mentioned by the Court.^51^ It is frequent in sexual assault cases that no autosomal STR profile from the sexual offender can be established from the collected genital swabs, due to male–female DNA mixtures with large amounts of female victim DNA, covering the genetic characteristics of the male perpetrator.^52^ We can therefore conclude that the necessary detection and prevention of recidivism by sex offenders cannot be achieved with standard autosomal STR profiles alone. The mildest measure to be more efficient in this regard seems to be the registration of an additional Y-STR profile from convicted sexual offenders in the NCODD.

It is noteworthy in this context that NCODDs that include Y-STR profiles already exist in several countries. Austria, for over ten years now, collects Y-STR profiles from crime scene traces and stores them in their NCODD, which has proven quite successful for connecting several sexual offences; Y-STR profiles of reference samples, however, are not generated and stored in the Austrian NCODD.^53^ A similar NCODD, for Y-STR trace profiles only, was established in Switzerland, starting in August 2023.^54^ In Italy, Y-STR profiles can already be registered on the NCODD^55^. Recently, Belgium passed a law amendment, laying down the legal grounds for the systematic generation of Y-STR profiles from convicted offenders in sexual assault cases and their inclusion in the NCODD. Y-STR profiles from other cases can be established and registered following an individual justification, based on case circumstances.^56^ In the Netherlands, Y-STR profiles from both trace material and reference samples can be stored in the NCODD based on a case-by-case assessment. These Y-STR profiles do not necessarily have to be linked to sexual offences. There are no restrictions in the Netherlands on registering Y-STR profiles in any type of criminal case if there is added value to answer the question at hand. However, in practice this is done mostly in serious cases such as sexual assault cases and violent crime.^57^ In Singapore, Y-STR profiles are generated and included in the NCODD of all reference samples of convicted offenders.^58^

In general, registering Y-STR profiles from convicted sex offenders on the NCODD would only affect a small portion of the male population, arguing in favor of the proportionality of such a measure. In Switzerland for example, of the 6039 individuals convicted of a violent offence in the year 2021, only 198 were convicted for rape or other sexual assault.^59^ This makes up only 3.3% of the larger category of violent crimes and, assuming that most of those convicts are male, only about 0.007% of the Swiss male population between 18 and 65 years old.^60^ An NCODD based on Y-STR profiles for convicted sexual offenders would therefore most likely not be classified as excessive by the Court.

However, just assuming that someone who has been convicted e.g., for a car theft would also be at a significantly increased risk —compared with the general population— for committing a sexual offense would violate the presumption of innocence. Sweepingly registering on the NCODD the Y-STR profile of all criminal offenders, convicted for all types of crimes, sexual or not, serious or not, with the purpose to identify quickly all those who will commit a sexual offense in the future, or have done so in the past, will therefore most likely not be admissible.

What might be admissible though is the inclusion of Y-STR profiles on the NCODD from violent offenders, convicted for a similarly serious crime as sexual offense, such as murder, in case such convicts would show a significantly higher prevalence for sexual offending. From the criminological literature we inspected, we were unable to find studies describing a solid association between violent offending and sexual offending, which, however, does not mean that such a relationship does not exist and will not be revealed, in case it does exist, in future studies.

### V.C. Registering Y-STR Profiles in NCODDs for the Purpose of Facilitating Familial Searching

In *Van der Velden*, the ECtHR stated that the fact that the offender demonstrated to be capable of committing a serious crime justified his inclusion in the NCODD.^61^ This connects the registration of a DNA profile to the conviction. Notably, this is not the case if a DNA profile is registered in the NCODD to identify relatives of the convicted criminal offender that are not registered in the NCODD. It is evident that we face a completely different situation when the storing of Y-STR profiles from convicted offenders in the NCODD is not bound to the detection and prevention of recidivism, but to the purpose of more efficient familial searching. In the second case, the primary purpose of storing a Y-STR profile of a convicted offender in the NCODD is not to re-identify this specific offender in cases of re-offending, but to find more readily his male relatives, in case they leave traces in any kind of potential future crimes, or left some in the past.

At the first glance, what seems to be primarily at stake here is not the privacy of the convicted offender, but the privacy of his male relatives that share more or less the same Y-STR information when it comes to standard Y-STRs. This is less relevant for RM Y-STRs, since they frequently differ already over only few generations. However, the standard Y-STRs with moderate mutation rates are primarily of interest for familial searching, exactly because they usually show the same alleles in all relatives including close and distant ones. Under the scenario of familial search, the reason for including Y-STR profiles in the NCODD is no longer to prevent re-offending of the convicted, but to identify family members of the unknown trace donor that will themselves be excluded as potential perpetrators through their non-matching autosomal STR profiles, but in turn can provide investigative leads to find the unknown perpetrator via Y-STR profile matching. This also raises ethical issues for society; because it might, e.g., reinforce general believes about criminality being ‘a family thing’.^62^

Concerning the familial search procedure through Y-STRs, it must be held that there are parallels with conventional familial searching through autosomal STR profiles. Familial searching, based on autosomal or Y-chromosomal data, comes with ethical challenges that have already widely been discussed in the literature, the biggest issue being the unequal probabilities for being included in a NCODD, due to racial bias in policing. In the US, it is, e.g., approximately four times more likely for African Americans to be identified through familial searching then for white offenders.^63^ While this problem has well to be acknowledged, we will not enter deeper into this debate here and instead focus on the general legal admissibility.

Familial searching in NCODDs based on autosomal data is practiced already in several countries with respective legislation. Familial searching through autosomal STR profiles, even though much less efficient because only successful for very close relatives, is also based on shared genetic information between individuals included in the NCODD (relatives of unknown sample donors) and individuals that are not included in the NCODD (unknown sample donors). Familial search via NCODDs based on autosomal STR profiles is legally permissive in a variety of countries, including, e.g., France, Germany, Sweden, Switzerland, the Netherlands, the UK or some states in the U.S.A.^64^ The main difference between using autosomal and Y-chromosomal STRs for familial search is the number of relatives that can be highlighted, which is more (close and distant ones) with Y-STRs and less (only very close ones) with autosomal STRs. From a law enforcement perspective, being able to highlight more potential relatives of an unknown perpetrator is advantageous as the more relatives and more different types of relatives can be revealed, the larger the chance that any of those is included in the NCODD to provide the wanted investigative leads. This argues in favor of the use of Y-STRs in NCODDs for familial searching from the perspective of law enforcement. We must, however, point out the fundamental difference between STRs on the autosomes and those on the Y-chromosome when it comes to the privacy of the respective genetic data: Whereas a (complete) autosomal STR profile will, with the exception of monozygotic twins, be found only in one single individual, i.e., the sample donor if a NCODD match is revealed, registering the Y-STR profile of a convicted offender on the NCODD most likely means registering the identical Y-STR profiles of his close and distant male relatives, when it comes to standard Y-STRs with moderate mutation rates. Shared DNA profiles between paternally related men challenge our prevailing concept of genetic privacy as an individual right and due to data shared through familial bonds, new concepts of genetic group privacy may arise in the future.^65^ However, given that group privacy is currently not much reflected in the prevailing law, the more pronounced sharing of genetic data between family members with Y-STR profiling does not seem to render a corresponding database inadmissible.

What seems more important here, under the currently established concept of privacy, is that by including Y-STR profiles in NCODDs, more personal data from the convicted offenders included in the NCODD are generated and registered. It could be argued that the additional Y-STR profile does not contain significantly more sensitive information than the autosomal STR profile that is already included in the NCODD. However, if the goal is to include Y-STR profiles in the NCODD for facilitating familial searching, more personal, i.e., genetic data from convicted offenders will be registered to facilitate the identification of offenders in cases and offenses that have nothing to do with the crime they have been registered for in the NCODD. In other words, the genetic data (Y-STR profile) is generated for a purpose that is not connected anymore to the offense the offender was previously convicted for. If it is not a sexual offender, the Y-STR profile will most likely not be necessary to re-identify him in case of re-offending, since his autosomal STR profile is already registered on the NCODD and is enough for re-identification. Therefore, the only valid argument for such an inclusion of Y-STR profiles from all convicted offenders in the NCODD, for which also autosomal STR profiles are already included in the NCODD, is a general increase in efficiency for police investigations (i.e., improving familial searching).^66^

However, a mere increase in efficiency of procedures for law enforcement is not a valid argument for the ECtHR to justify the restriction of the fundamental right to privacy in a broad sense. The most efficient way to identify perpetrators through DNA would be a universal DNA database, holding autosomal STR profiles from the entire national population. The Court repeatedly dismissed the ‘efficiency argument’ in relation to hypothetical universal databases in the past.^67^

A Y-STR database for convicted offenders is not a universal database. However, in its effect it might get close to a universal database depending on the Y-chromosomal population substructure in the country and the number of collected men included in the NCODD. China has chosen the strategy of collecting Y-STR profiles from the general population to store them in a database, which notably *is not* a NCODD as we are discussing in this article. It can be assumed that Chinese authorities have registered already more than 30 million Y-STR profiles in their database. In the case of the province Henan, about 10% of the general local male population has been typed for Y-STRs and their Y-STR profiles are stored in the database, leading to a coverage of over 98% of the male population through paternal family trees.^68^

Let us extrapolate this to Europe through the example of the UK, even though the Y-chromosomal population structure likely is different and less detailed genealogical records might be available in the UK: The UK’s NCODD holds 5.6 million entries in 2020, 80% of them from men.^69^ This amounts to 4.5 million registered male individuals, what would be equivalent to over 13% of the entire UK male population.^70^ If for every one of those male criminal offenders in the NCODD, a Y-STR profile would be established and stored in the UK’s NCODD, we can assume that over 95% of the entire male population of the UK could result in a match with standard Y-STRs in the NCODD. The registration of Y-STR profiles for all convicted offenders could therefore result in what seems a quasi-universal database for the male population in countries with a very larger forensic DNA database, such as the UK.^71^

Universal DNA databases are deemed widely disproportionate in Europe, and would most likely not be accepted by the ECtHR.^72^ We can therefore assume that such a comprehensive registration of Y-STR profiles from all convicted offenders in the NCODD, irrespective of the type of recordable crime committed, which under certain circumstances may parallel in its effect a universal DNA database for men, would not be deemed justified under Article 8 ECHR.

However, as we already mentioned in the previous chapter, the situation is different when only Y-STRs of sexual offenders are included in the NCODD. Let us illustrate this again with the outstanding example of the very large NCODD in the UK: In England and Wales, there were 60,000 registered sex offenders in 2019.^73^ Convicted sex offenders are usually also registered on the NCODD. Therefore, we may assume as a realistic figure, 80,000 DNA entries from sex offenders from the whole UK including Scotland, representing only about 0.2% of the entire male population of the UK.^74^ If Y-STR profiles would be established from those 80,000 men for the purpose of detecting and preventing recidivism and stored in the NCODD, this remains quite far from a comprehensive registration of male lineages that indirectly comes close to a universal database. This would largely hold true even if those 80,000 Y-STR profiles would be used not only for their initial purpose of identifying recidivists, but also for familial searching.

### V.D. Is a NCODD Based on Y-STRs Discriminatory under Article 14 ECHR?

In *Marper*, the Court did not assess the question, whether the retention of DNA profiles from non-convicted individuals was discriminatory in the sense of article 14 of the convention.^75^ However, it is well possible that article 14 could be violated by a comprehensive Y-STR database that systematically registers more genetic data from all male offenders than from female offenders. Such an approach could well be considered discriminatory because its creation is exclusively based on sex, i.e., male sex. It might however be justifiable if only male sex offenders were included with their Y-STR profiles in the NCODD for two reasons: (i) sex offenders are mostly men and rarely women and (ii) the particular circumstances of the case justify an inequality of treatment. In sexual assault cases, as described above, a lot of female victim DNA and only little amounts of male perpetrator DNA are typically detected. Furthermore, if a Y-STR profile is generated from a vaginal swab after sexual assault, it is fair to assume that the Y-STR profile is indeed related to a physical contact and therefore likely to the sexual offence, since DNA transfer to the vaginal or anal regions other than through direct contact is not very probable in such protected regions of the body. Therefore, also under article 14, registering Y-STR profiles from sex offenders in the NCODD will most likely be justifiable, while registering Y-STR profiles from all convicted men might not. However, given the lack of precedents, for us it seems tantamount to an oracle to predict the Court’s jurisdiction on this issue under article 14 ECHR.

## VI. CONCLUDING REMARKS

Expanding NCODDs with Y-STR profiles is expected to allow solving more criminal cases with male perpetrators for whom autosomal STR profiles cannot be obtained because of male:female DNA mixtures, particularly sexual assault cases, than can be solved with NCODDs solely based on autosomal STRs, thereby providing benefits for law enforcement. Expanding NCODDs with Y-STR profiles can serve two main purposes: (i) re-identifying offenders in the NCODD for whom no autosomal STR profile is available in the new case, which mostly applies to sexual assault causes with male–female mixed DNA evidence and (ii) tracing unknown male perpetrators not yet included in the NCODD through identifying their relatives in the NCODD based on familial searching to find unknown perpetrators, which is more effective with Y-STR profiles than it is with autosomal STR profiles. From a technical point of view, especially with the latest developments on commercial Y-STR kits with expanded number of markers for increased male lineage differentiation, and the latest developments on RM Y-STRs for increased male relative differentiation, we believe that now for the first time it has become feasible to use Y-STRs in this way. However, such Y-STR uses involving NCODDs raise valid concerns about genetic privacy. The effectiveness and ethically and societally responsible use of Y-STR profiles in NCODDs will depend on how it is practically implemented. Focusing on a European perspective, our analysis of the fundamental rights framework of the CoE prompts us to believe that a Y-STR-based NCODD for the prime purpose of re-identifying convicted sexual offenders would be in line with the case law of the ECtHR, whereas a general inclusion of Y-STR profiles in NCODDs from all convicted offenders and for the prime purpose of familial searching may not. With this paper, we intend to stimulate a wide discussion between the various stakeholders on the potential expansion of criminal offender DNA databases with Y-STR profiles. We believe that it is important for forensic geneticists, bioethicists, policymakers, law enforcement agencies and other stakeholders to engage in thoughtful discussions to carefully weigh the benefits and risks of the enhanced use of Y-STRs for criminal investigations via expanding NCODDs with Y-STR profiles, which in some countries has already been implemented in forensic practise.

## Footnotes

1 Manfred Kayser et al., *Evaluation of Y-chromosomal STRs: a multicenter study*, 110(3) Int. J. LegalMed. 125 (1997).

2 Manfred Kayser, *Forensic use of Y-chromosome DNA: a general overview*, 136(5) Hum. Genet. 621 (2017).

3 Peter Gill et al., *Genotyping and interpretation of STR-DNA: Low-template, mixtures and database matches—Twenty years of research and development,* 18 ForensicSci. Int. Genet. 100 (2015); Mechthild Prinz et al., Validation and casework application of a Y chromosome specific STR multiplex, 120(3) Forensic Sci. Int. 177 (2001).

4 The possibility to narrow down suspect pools emerging from familial searching or DNA mass testing based on the analysis of lineage markers (Y-STRs and mitochondrial DNA sequencing) is e.g., explicitly foreseen for in the Swiss law (Article 9a(b), Swiss DNA Profiles Act, SR363, in conjunction with the Message on the amendment of the Swiss DNA Profiles Act of Dec. 04, 2020, BBl 2021 44, 38). A similar regulation for candidate lists from familial searching can be found in Article 44octies of the Belgian Act amending the Code of Criminal Procedure, the Act of 22 March 1999 on the procedure for identification by DNA analysis in criminal matters and the Act of 5 August 1992 on the police service of March 07, 2024, M.B. 14 mars 2024, p. 31939.

5 Atif Adnan et al., *Improving empirical evidence on differentiating closely related men with RM Y-STRs: A comprehensive pedigree study from Pakistan*, 25 ForensicSci. Int. Genet. 45 (2016).

6 Manfred Kayser et al., *Relating two deep-rooted pedigrees from Central Germany by high-resolution Y-STR haplotyping*, 1(2) Forensic Sci. Int. Genet. 125 (2007).

7 Manfred Kayser & Peter de Knijff*, Improving human forensics through advances in genetics, genomics and molecular biology*, 12(3) Nat. Rev. Genet. 179 (2011); Kayser, *supra* note 2; Angie Ambers et al., *Improved Y-STR typing for disaster victim identification, missing persons investigations, and historical human skeletal remains*, 132 Int. J. LegalMed. 1545 (2018).

8 Mannig A. Connors, *DNA databases: The case for the Combined DNA Index System*, 29 WakeForest L. Rev. 889 (1994).

9 Emily West & Vanessa Meterko, *Innocence project: DNA exonerations, 1989–2014: review of data and findings from the first 25 years*, 79 Alb. L. Rev. 717 (2015).

10 Barbara Prainsack & Victor Toom, *Performing the Union: The Prüm Decision and the European dream*, 44(1) Stud. Hist. Philos. Biol. Biomed. Sci. 71 (2013).

11 Jianye Ge & Bruce Budowle, *Forensic investigation approaches of searching relatives in DNA databases*, 66(2) J. ForensicSci. 430 (2021).

12 Kaye N. Ballantyne et al., Mutability of Y-chromosomal microsatellites: rates, characteristics, molecular bases, and forensic implications, 87(3) Am. J. Hum. Genet. 341 (2010); Arwin Ralf et al. *Identification and characterization of novel rapidly mutating Y-chromosomal short tandem repeat markers*, 41(9) Hum. Mutat. 1680 (2020).

13 Arwin Ralf et al., *Large-scale pedigree analysis highlights rapidly mutating Y-chromosomal short tandem repeats for differentiating patrilineal relatives and predicting their degrees of consanguinity*, 142(1) Hum. Genet. 145 (2023).

14 Jonelle M. Thompson et al., *Developmental validation of the PowerPlex® Y23 System: a single multiplex Y-STR analysis system for casework and database samples*, 7 (2) ForensicSci. Int. Genet. 240 (2013); Siddhita Gopinath et al., *Developmental validation of the Yfiler® Plus PCR Amplification Kit: An enhanced Y-STR multiplex for casework and database applications*, 24 ForensicSci. Int. Genet. 164 (2016).

15 Cesare Rapone, *Forensic genetic value of a 27 Y-STR loci multiplex (Yfiler® Plus kit) in an Italian population sample*, 21 ForensicSci. Int. Genet. e1 (2016).

16 Maarten H.D. Larmuseau et al., *A substantially lower frequency of uninformative matches between 23 versus 17 Y-STR haplotypes in north Western Europe*, 11 ForensicSci. Int. Genet. 214 (2014).

17 Kaye N. Ballantyne et al., *Toward male individualization with rapidly mutating y-chromosomal short tandem repeats.* 35(8) Hum. Mutat. 1021 (2014).

18 Ralf, *supra* note 13.

19
*Id.*

20 Arwin Ralf et al., *RMplex: An efficient method for analyzing 30 Y-STRs with high mutation rates*, 55 ForensicSci. Int. Genet. 102,595 (2021).

21 Ralf, *supra* note 13.

22 Connor, *supra* note 8.

23 Pieter E. Baay et al., *Ex-imprisoned homicide offenders: Once bitten, twice shy?*, 16.3 HomicideStud. 259 (2012).

24 Depending on the legislation, the exact definitions of rape and sexual assault may vary. If we refer in the following to sexual assault this wording means to include rape in the form of penile penetration, but also all other forms of serious offense against sexual integrity.

25 Eva Mulder et al., *Recidivism in subgroups of serious juvenile offenders: Different profiles, different risks?*, 22(2) Crim. Behav. Ment. Health. 122 (2012).

26 Brian A. Reaves, *Felony defendants in large urban counties, 2009-statistical tables,* US Department ofJustice, Bureau ofJusticeStatistics (2013), https://bjs.ojp.gov/library/publications/felony-defendants-large-urban-counties-2009-statistical-tables (accessed June 05, 2023).

27 Keith Soothill et al., *Sex offenders: Specialists, generalists - Or both? A 32-year criminological study*, 40(1) Brit. J. Criminol. 56 (2000).

28 Mary P. Koss, *Restoring Rape Survivors: Justice, Advocacy, and a Call to Action*, 1087(1) Ann. N. Y. Acad. Sci. 206 (2006).

29 Alessandra Iuvaro et al., *Male DNA under female fingernails after scratching: transfer and persistence evaluation by RT-PCR analysis and Y-STR typing*, 132 Int. J. LegalMed. 1603 (2018)

30 Nederlands Forensisch Instituut, Nieuws, https://www.forensischinstituut.nl/actueel/nieuws/2022/12/24/dna-specialisten-van-het-nfi-leveren-bijdrage-aan-tientallen-buitenlandse-strafzaken/ (accessed Aug. 28, 2023).

31 Nederlands Forensisch Instituut, Magazine, https://magazines.forensischinstituut.nl/atnfi/2022/38/zonder-verwantschapsonderzoek-hadden-we-de-moordenaar-van-milica-van-doorn-nooit-gevonden (accessed Aug. 28, 2023).

32 Marieke van de Rakt, Paul Nieuwbeerta & Nan Dirk de Graaf, *Like father, like son: The relationships between conviction trajectories of fathers and their sons and daughters*. 48(4) Br. J. Criminol. 538 (2008).

33 Ralf et al., *supra* note 13, at 154.

34 Adri Vermaat, Doorbraak door DNA in de zaak-Vaatstra Groot onderzoek leidt naar verdachte in naburig dorp, Trouw, Nov. 202,012, at 4.

35 Ge & Budowle, *supra* note 11, at 433.

36 Lutz Roewer et al., *DNA commission of the International Society of Forensic Genetics (ISFG): Recommendations on the interpretation of Y-STR results in forensic analysis*, 48 ForensicSci. Int. Genet. 102,308 (2020).

37 Amke Caliebe & Michael Krawczak, *Match probabilities for Y-chromosomal profiles: A paradigm shift,* 37 ForensicSci. Int. Genet. 200 (2018).

38 YHRD: Y-Chromosome STR Haplotype Reference Database, https://yhrd.org/pages/resources/stats (accessed Aug. 28, 2023).

39 Mikkel M. Andresen & David K. Balding, *How convincing is a matching Y-chromosome profile?*, 13(11) PLoS Genet. e1007028 (2017).

40 Such an approach for DNA mass testing was chosen by the Swiss legislator in article 256(1) of the Swiss Criminal Procedure Code, SR 312.0.

41 Christi J. Guerrini et al., *Four misconceptions about investigative genetic genealogy,* 8(1) J. LawBiosci. lsab001, 1, 3 (2021); The Intercept, https://theintercept.com/2023/08/18/gedmatch-dna-police-forensic-genetic-genealogy (accessed Oct. 18, 2023).

42 Peter de Knijff, *From next generation sequencing to now generation sequencing in forensics*, 38 ForensicSci. Int. Genet. 175–180 (2019).

43 Øyvind Bleka et al., *An examination of STR nomenclatures, filters and models for MPS mixture interpretation*, 48 ForensicSci. Int. Genet. 102,319 (2020).

44 UN Educational, Scientific and Cultural Organization (UNESCO), *International Declaration on Human Genetic Data*, 16 October 2003, Article 12.

45
*Van der Velden v. the Netherlands*, appl. no. 29514/05 (dec.) ECtHR (Dec. 7, 2006); *S and Marper v. the United Kingdom*, appl. Nos. 305,621/04 and 30,566/04 ECtHR [GC] paras. 66, 67 (Dec. 4, 2008); *Peruzzo and Martens v. Germany*, appl. Nos. 7841/08 and 57,900/12 (dec.) ECtHR (June 04, 2013), para. 33; *Aycaguer v. France*, appl. no. 8806/12 ECtHR (June 22, 2017), para. 33; *Gaughran v. the United Kingdom*, appl. no. 45245/15 ECtHR (Feb. 13, 2020), para. 63.

46 Oliver M. Tuazon, *Universal forensic DNA databases: acceptable or illegal under the European Court of Human Rights regime?* 8(1) J. LawBiosci. lsab022, 1, 5 (2021).

47
*Adamson v. the United Kingdom*, appl. no. 42293/98 (dec.) ECtHR (Jan. 26, 1999); *Van der Velden; Gardel v. France*, appl. no. 16428/05 ECtHR (Dec. 17, 2009) paras. 63–64; *Trajkovski and Chipovski v. North Macedonia*, appl. Nos. 53,205/13 and 63,320/13 ECtHR (Feb. 13, 2020) para. 51.

48
*Gaughran*, at para. 76; *M.K. v. France*, appl. no. 19522/09 ECtHR (April 18, 2013), paras. 33 and 39; *Peruzzo and Martens*, at para. 44.

49
*X and Y v. the Netherlands,* appl. no. 8978/80 ECtHR (Mar. 26, 1985), paras. 23 and 27; *Stubbings and others v. the United Kingdom*, appl. Nos. 22,083/93 and 22,095/93 ECtHR (Oct. 22, 1996), para. 64; *Adamson*; *Gardel*, at paras. 63 and 69.

50
*Van der Velden.*

51
*S and Marper*, at para. 75; *Gaughran*, at para. 81.

52 Josephine Purps, Maria Geppert, Marion Nagy, Lutz Roewer, *Validation of a combined autosomal/Y-chromosomal STR approach for analyzing typical biological stains in sexual-assault cases*, 19 ForensicSci. Int. Genet. 238 (2015).

53 Franz Neuhuber, Eva Klausriegler, Gabriele Kreindl, Waltraud Zahrer, Bettina Dunkelmann, Ines Pickrahn, Jan Cemper-Kiesslich, *The efficiency of Y-chromosome markers in forensic trace analysis and their inclusion in the Austrian National DNA Database*, 4(1) ForensicSci. Int. Genet. Suppl. Series, e172 (2013).

54 Swiss DNA Profiles Act, Amendment (De. 17, 2021), BBl 2021 2998.

55 Forensic Science Regulator Guidance on Y-STR Profiling, FSR-G-227, Issue 1, 8 (2021), https://assets.publishing.service.gov.uk/government/uploads/system/uploads/attachment_data/file/973580/227_Y_STR_guidance__Issue1.0_Final.pdf (accessed June 05, 2023).

56 Article 8, Belgian Act amending the Code of Criminal Procedure, the Act of 22 March 1999 on the procedure for identification by DNA analysis in criminal matters and the Act of 5 August 1992 on the police service of March 07, 2024, M.B. 14 mars 2024, p. 31939. For a short summary of the amendment (in French) see this news article on polinfo.be of March 15, 2024: *Analyse ADN en matière pénale: extension de l’échange international de profils ADN, allongement du délai de conservation des échantillons de référence et possibilité de recherche familiale,*https://polinfo.kluwer.be/NewsView.aspx?contentdomains=POLINFO&id=VS301078940&lang=fr*(accessed July 03, 2024).*

57 Personal communication with Arnoud Kal from the Netherlands Forensic Institute (Aug. 242,023).

58 Ge & Budowle, *supra* note 11.

59 Swiss Federal Statistical Office, *Convictions of adults in 2021*, https://www.bfs.admin.ch/bfs/de/home/statistiken/kriminalitaet-strafrecht/strafjustiz.gnpdetail.2022-0375.html (accessed May 24, 2023)

60 Swiss Federal Statistical Office, *Permanent resident population by age, sex and category of citizenship, 2010–2021*, https://www.bfs.admin.ch/asset/de/je-d-01.02.03.02 (accessed May 24, 2023)

61
*Van der Velden*.

62 Erica Haimes, *Social and Ethical Issues in the Use of Familial Searching in Forensic Investigations: Insights from Family and Kinship Studies,* 34 J.L. Med. & Ethics 263,270 (2006); Jessica D. Gabel, *Probable Cause from Probable Bonds: A Genetic Tattle Tale Based on Familial DNA*, 21(1) HastingsWomen’s L. J. 3, 45 (2010). Indeed, there is evidence supporting the notion that crime is more prevalent in some families than it is in others, what could argue in favor of the efficiency of familial searches. See, e.g., Thomas Frisell, Paul Lichtenstein & Niklas Långström*, Violent crime runs in families: a total population study of 12.5 million individuals.* 41(1) Psychol. Med. 97 (2011); Anu Putkonen et al., *Transmission of violent offending and crime across three generations.* 42(2) Soc. PsychiatryPsychiatr. Epidemiol. 94 (2007); van de Rakt, *supra* note 33. However, the reason for this has most probably nothing to do with genetics, but most likely with the common social environment. Therefore, any faulty and simplistic association between genetics and prevalence to commit crime should be avoided, to prevent stigmatizations.

63 Gabel, *supra* note 60, 45; Henry T. Greely et al., *Family ties: The use of DNA offender databases to catch offenders’ kin*, 34(2) J. LawMed. Ethics,:248, 258 (2006); Simon A. Cole, *How much justice can technology afford? The impact of DNA technology on equal criminal justice*, 34(2) Sci. PublicPolicy, 95, 102 (2007); Brett Mares, *A chip off the old block: Familial DNA searches and the African American community*, 29(2) LawInequal. 395, 407 (2011); Katharina Clausius, Erin Kenny and Michael J. Crawford, *BILL S-231: The Ethics of Familial and Genetic Genealogical Searching in Criminal Investigations*, 6(3–4) Can. J. Bioeth. 44, 46 (2023).

64 Henrik Westermark et al., *The regulation of the use of DNA in law enforcement*, *current to: 28.08.2020, E-AVIS ISDC 2020–02,*SwissInstitute ofComparativeLaw 1,9 (2020), https://www.isdc.ch/media/1953/e-2020-02-20-016-use-of-dna.pdf (accessed June 02, 2023).

65 Dara Hallinan, Paul de Hert, *Genetic Classes and Genetic Categories: Protecting Genetic Groups through Data Protection Law.*GroupPrivacy. New challenges of data technologies (Linnet Taylor, Luciano Floridi, Bart van der Sloot, eds.), PhilosophicalStudiesSeries 126, 175 (Springer, Cham, 2017); Róisín Á Costello, *Genetic Data and the Right to Privacy: Towards a Relational Theory of Privacy?* 22 Hum. RightsLawRev. 1 (2022).

66 Notably, this argument does not apply to cases of sexual offense where autosomal STR profile could not be generated because of the problem of male–female DNA mixture and the purpose of storing Y-STR profiles in the NCODD is re-identification via Y-STR profiles, which in such cases is impossible with autosomal STRs because autosomal STR profiles are not available from the evidence DNA (see section V.2).

67
*M.K.*, at para. 37; *Aycaguer*, at para. 34; *Gaughran*, at para. 89.

68 Emile Dirks & James Leibold, *Genomic surveillance – Inside China’s DNA dragnet*, Australian Strategic Policy Institute, Policy Brief Report No. 34/2020, 1, 5 and 8 (2020) https://s3-ap-southeast-2.amazonaws.com/ad-aspi/2020-06/Genomic%20surveillance_1.pdf?QhPFyrNVaSjvblmFT24HRXSuHyRfhpml (accessed June 05, 2023).

69 UK National DNA Database Strategy Board, Biennial Report 2018–2020, https://assets.publishing.service.gov.uk/government/uploads/system/uploads/attachment_data/file/913015/NDNAD_Strategy_Board_AR_2018-2020_print.pdf (accessed June 05, 2023).

70 UK Office for National Statistics, *Estimates of the population for the UK, England, Wales, Scotland and Northern Ireland*, (Dec. 21, 2022), https://www.ons.gov.uk/peoplepopulationandcommunity/populationandmigration/populationestimates/datasets/populationestimatesforukenglandandwalesscotlandandnorthernireland (accessed June 05, 2023).

71 However, it needs to be noted that many of such matches would link the sample donor to a distant paternal relative, as can be shown with RM Y-STRs, which would in practice not readily lead to the identification of the perpetrator (as was described above). Moreover, weather this calculation example holds true in practice, or not, depends on various unknown factors regarding the country’s Y-chromosomal population substructure and the representation of male lineages in the NCODD, which is expected to differ between different countries based on differences in male population history, male reproductive success, and various other biological and non-biological factors.

72 Tuazon, *supra* note 15, at 23–24; *Gaughran*, at para. 89.

73 His Majesty’s Inspectorate of Probation, *Sexual Offending*, https://www.justiceinspectorates.gov.uk/hmiprobation/research/the-evidence-base-probation/specific-sub-groups/sexual-offending/ (accessed Aug. 09, 2023)

74 The 20,000 entries added for Scotland were simply extrapolated from the relative population size.

75
*S and Marper*, at para. 129.

